# The Complete Chloroplast Genome of a Vietnam Endemic Aroid, *Homalomena perplexa* K.Z.Hein, Vuong, Bao & V.S.Dang, and Updated Comparative Genomics in Araceae

**DOI:** 10.1002/ece3.73189

**Published:** 2026-02-27

**Authors:** Nhat Nam Nguyen, Ngoc Trai Nguyen, Hoang Dang Khoa Do

**Affiliations:** ^1^ School of Agriculture and Aquaculture Tra Vinh University Vinh Long Province Vietnam; ^2^ Functional Genomics Research Center, NTT Hi‐Tech Institute Nguyen Tat Thanh University Ho Chi Minh City Vietnam; ^3^ Nguyen Tat Thanh University Center for Hi‐Tech Development, Saigon Hi‐Tech Park Ho Chi Minh City Vietnam

**Keywords:** aroid, Aroideae, infrafamilial relationship, phylogeny, plastome evolution

## Abstract

*Homalomena perplexa* K.Z.Hein, Vuong, Bao & V.S.Dang has been recently recognized and identified as a Vietnamese endemic species. Its chemical contents and bioactivities have been investigated, but its genomic information is still scarce. In the current study, we sequenced and characterized the complete chloroplast genome of *H. perplexa* using the next‐generation sequencing method. The circular genome was 169,893 bp in length and contained 79 protein‐coding genes, 30 transfer RNA genes, and four ribosomal RNA genes. Nucleotide diversity analysis revealed only two hypervariable regions between the two *Homalomena* chloroplast genomes. However, repeat analysis indicated that there were shared and unique repeats between *H. perplexa* and *H. occulta* chloroplast genomes. In Araceae chloroplast genomes, there was a high variation of the junctions among the large‐single copy, small‐single copy, and inverted repeat regions, especially 13 variations identified in Aroideae species. In addition, phylogenetic analysis revealed the monophyly of Araceae subfamilies and a close relationship between *Homalomena* and *Furtadoa* genera. The results of this study enlarge the genomic data of the *Homalomena* genus and provide useful information for further comparative genomic studies of *H. perplexa* and related species in Aroideae. In addition, updates on comparative chloroplast genomics and phylogenetic relationships add valuable information for further evolutionary studies of Araceae.

## Graphical abstract

The complete chloroplast genome of *Homalomena perplexa*, an endemic aroid to Vietnam, was completely sequenced and characterized. In addition, comparative genomic analysis revealed junction variations and phylogenetic analysis confirmed the infrafamilial relationships of Araceae.

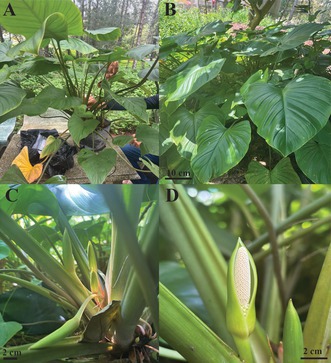

## 1 Introduction

*Homalomena* Schott, a member of the Araceae family, contains 163 accepted species that are distributed in South Asia, East Asia, Southeast Asia, and Melanesia (Plants of the World Online 2025). Recently, various new *Homalomena* species have been identified, including *Homalomena renda*, *Homalomena belitungensis* A.S.D.Irsyam & M.R.Hariri from Indonesia, *Homalomena punctifolia* K.Z.Hein and Naive and *Homalomena sigmoidea* K.Z.Hein and Naive from Myanmar, and *Homalomena perplexa* K.Z.Hein, Vuong, Bao & V.S.Dang from Vietnam (Dang et al. 2023; Hein and Naive 2024; Hariri and Irsyam 2025; Irsyam et al. 2025). These new species indicate a potentially high biodiversity of the *Homalomena* genus and need further investigations. In addition, different compounds such as monoterpenes and linalool have been identified in *Homalomena* species extraction, which exhibited antimicrobial, antifungal, and anti‐inflammatory activities (Rozman et al. 2018; Dam and Van 2022; Nguyen, Doan, et al. 2023; Nguyen, Nguyen, et al. 2023; Gupta et al. 2025; Tamang et al. 2025). In the case of *H. perplexa* , the essential oil extraction from leaves and rhizomes contained various compounds (e.g., sabinene, limonene, and linalool) which displayed antibacterial activities (Truong, Nguyen, et al. 2025; Truong, Van, et al. 2025). Although high diversity and pharmacological features of *Homalomena* have been intensively studied, the genomic data (e.g., complete organelle and nuclear genomes) of *Homalomena* species are still scarce. Therefore, more genomic studies examining *Homalomena* species should be conducted.

Chloroplast is an essential organelle of land plants, which contains a circular genome (consisting of a large‐single copy (LSC), a small‐single copy (SSC), and two identical inverted repeat (IR) regions) encoding genes responsible for photosynthesis (Dobrogojski et al. 2020). Within the Araceae family, several complete chloroplast genomes have been reported (Abdullah, Henriquez, Mehmood, Shahzadi, et al. 2020; Henriquez et al. 2020; Abdullah et al. 2021; Li et al. 2025). These chloroplast genomes revealed polymorphic loci, repeat content, and genomic features across aroid species. In addition, the chloroplast genome provided useful data for mining molecular markers, exploring selective pressure, and phylogenetic relationships among Araceae species (Li et al. 2022). However, there has not been a comprehensive study examining whole chloroplast genomes of all Araceae subfamilies so far. Although there are more than 100 accepted *Homalomena* species, only one complete chloroplast genome of *Homalomena occulta* has been reported (Zhang et al. 2021). The lack of genomic data limits the exploration of evolutionary history among *Homalomena* species. Therefore, in the current study, we sequenced and characterized the complete chloroplast genome of *H. perplexa* , an aroid species endemic to Vietnam. This study aims to (1) compare the chloroplast genome features between *H. perplexa* and *H. occulta*, focusing on genone structure, gene content, and repeat composition, (2) locate hypervariable regions in *Homalomena* chloroplast genomes, (3) explore variations of junctions between LSC, SSC, and IR regions in Araceae chloroplast genomes, and (4) reconstruct phylogenetic relationships among Araceae subfamilies based on 79 protein‐coding regions. The results of this study provide new insights into chloroplast genome features of *Homalomena* species and an update on comparative chloroplast genome analysis of Araceae.

## 2 Materials and Methods

### 2.1 Plant Material, DNA Extraction, and Sequencing

**FIGURE 1:**
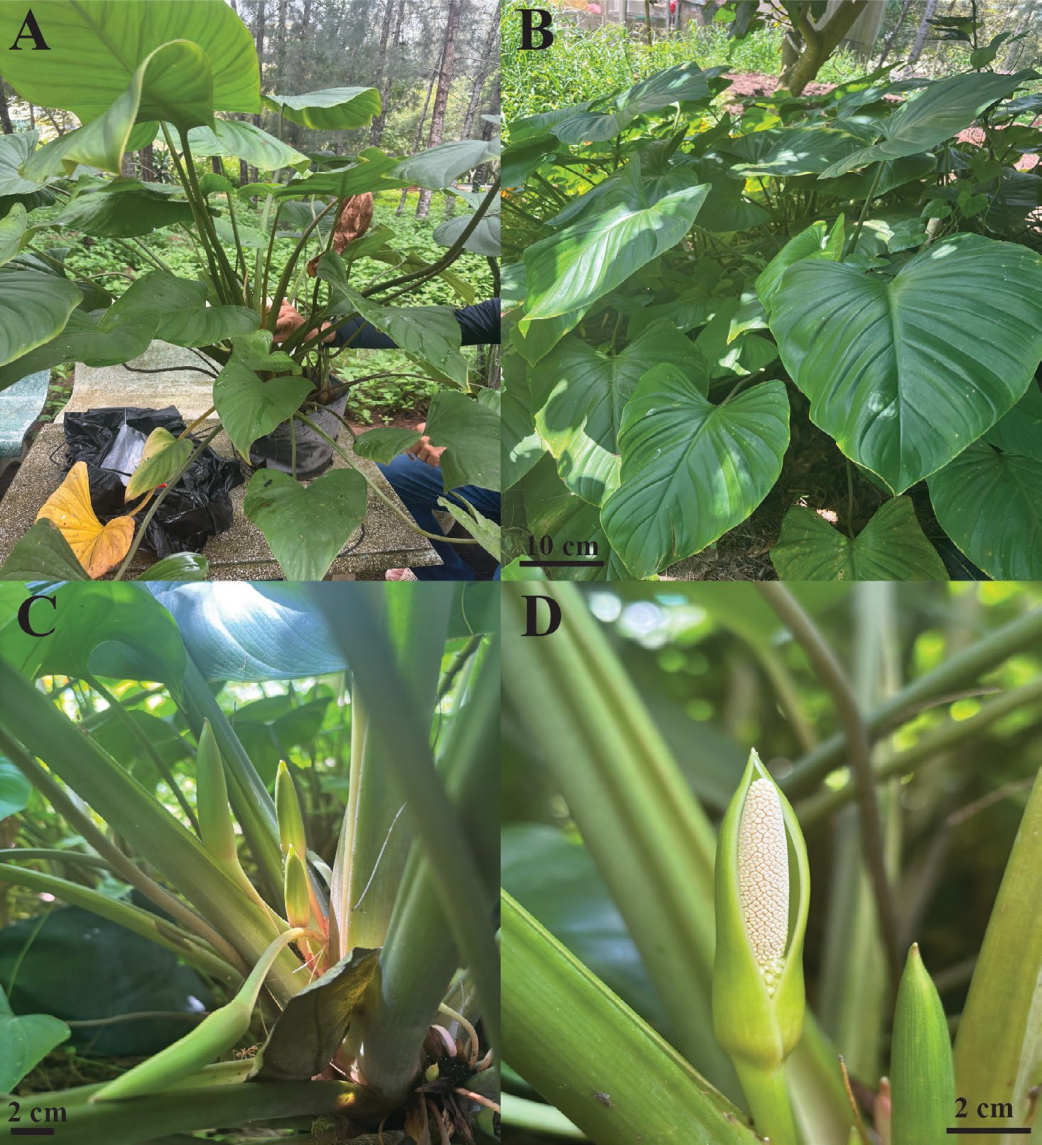
Photos of *Homalomena perplexa*. (A) Whole plant collected at Con Dao, Ho Chi Minh City. (B) Whole plant transplanted at Tra Vinh University, Vinh Long province. (C) Blooms with slender peduncles and green spathes. (D) Bloom at pistillate anthesis. The photos were taken by Nhat Nam Nguyen.

The whole plant of *H. perplexa* was transplanted from Con Dao, Ho Chi Minh City to Tra Vinh University, Vinh Long province (Figure 1). The living sample of *H. perplexa* was available at Tra Vinh University, Vinh Long province (contact person: Nguyen Nhat Nam, nnnam@tvu.edu.vn). No permission is required to collect the sample of *H. perplexa* . A healthy leaf was collected and dried using silica gel beads. The dried sample was stored in a deep freezer at −81°C before being used to extract total genomic DNA using Dneasy Plant Pro Kit (Qiagen, USA). The quality of DNA sample was checked using a NanoDrop One Microvolume UV–Vis Spectrophotometer (Thermo Fisher Scientific, USA) and 1% agarose gel electrophoresis. The DNA sample which had a clear band on agarose gel and a concentration over 100 ng/μl was used to prepare a library for sequencing using a Native Barcoding Kit 24 V14 (SQK‐NBD114.24, Oxford Nanopore Technologies, UK) following the manufacturer's protocol. The library was then sequenced using a PromethION flow cell (R10.4.1, FLO‐PRO114M) and a PromethION 2 Solo device, monitored by MINKNOW v24.11.10 with a High Accuracy option for the basecalling process. The quality of output reads in fastq format was checked using nanoplot v1.41.6 and then filtered using Filtlong v0.3.1 (https://github.com/rrwick/Filtlong) to remove reads that are shorter than 1000 bp and have a Qscore lower than 8 (De Coster and Rademakers 2023). Consequently, among 424,520 raw reads (accounting for 2.08 GB), 313,902 reads (composing 1.87 GB) were kept for further analyzes.

### 2.2 Genome Assembly and Annotation

The filtered reads were assembled to complete the chloroplast genome of *Homalomena occulta* (MW145396) to isolate chloroplast‐related reads using minimap2 with default settings (Li 2018). Then, the isolated reads were assembled using Flye v2.9.6 with the settings of ‐‐nano‐corr, ‐i 10, ‐g 0.17 m, and ‐t 60 to complete the chloroplast genome of *H. perplexa* (Kolmogorov et al. 2019). The complete chloroplast genome of *H. perplexa* was annotated using GeSeq (https://chlorobox.mpimp‐golm.mpg.de/geseq.html) with the default settings for the chloroplast genome (Tillich et al. 2017). The annotation of protein‐coding genes and transfer RNAs was confirmed using Geneious Prime v2025.0 for start and stop codons and tRNAscan‐SE, respectively (Chan and Lowe 2019). The map of the chloroplast genome was illustrated using OrganellarGenomeDRAW (OGDRAW) version 1.3.1 with default settings (Greiner et al. 2019). The complete chloroplast genome of *H. perplexa* was submitted to the GenBank database under accession number PX569150.

### 2.3 Comparative Chloroplast Genome Analyzes

For finding small sequence repeats (SSRs) in two chloroplast genomes of *Homalomena* species, Phobos embedded in Geneious Prime v2025.0 with a minimum threshold of 10, 12, 15, 20, and 24 bp for mononucleotide repeats, dinucleotide repeats, trinucleotide repeats, pentanucleotide repeats, and hexanucleotide repeats, respectively. Similarly, long repeats (e.g., forward, complement, reverse, and palindromic types) were located using the “Find Repeat” function in Genenious Prime v2025.0 with a minimum length of 20 bp. Nucleotide diversity analysis among two chloroplast genomes of *H. occulta* and *H. perplexa* was conducted using DnaSP 6 with a window length of 1000 and a step size of 200 (Rozas et al. 2017). The junctions among the large‐single copy, small‐single copy, and inverted repeat regions were located using IRplus and manually illustrated for 46 available complete chloroplast genomes, representing 45 genera of Araceae (Table 1) (Díez Menéndez et al. 2023).

**TABLE 1:** List of species for comparative genomic and phylogenetic analyzes.

Family	Subfamily	Species	Accession number
Araceae	Aroideae	*Alocasia fornicata*	MK636779
	Aroideae	*Aglaonema commutatum*	OR068727
	Aroideae	*Amorphophallus titanum*	MN046883
	Aroideae	*Anchomanes hookeri*	MN551188
	Aroideae	*Anubias heterophylla*	MN046884
	Aroideae	*Arisaema bockii*	MZ380241
	Aroideae	*Arisarum simorrhinum*	MN046886
	Aroideae	*Caladium lindenii*	ON707033
	Aroideae	*Calla palustris*	MN046887
	Aroideae	*Carlephyton glaucophyllum*	MT161478
	Aroideae	*Colocasia esculenta*	OP589403
	Aroideae	*Cryptocoryne striolata*	OM912764
	Aroideae	*Dieffenbachia seguine*	KR262889
	Aroideae	*Furtadoa mixta*	PQ539039
	Aroideae	*Hayarum mirispathum*	PQ772791
	Aroideae	*Homalomena occulta*	MW145396
	Aroideae	*Homalomena perplexa*	PX569150
	Aroideae	*Leucocasia gigantea*	MN972442
	Aroideae	*Montrichardia arborescens*	MN046889
	Aroideae	*Philodendron hederaceum*	OM937109
	Aroideae	*Pinellia pedatisecta*	OR772809
	Aroideae	*Pistia stratiotes*	MN885890
	Aroideae	*Sauromatum giganteum*	MN626718
	Aroideae	*Schismatoglottis calyptrata*	MN046892
	Aroideae	*Steudnera colocasiifolia*	MT161479
	Aroideae	*Stylochaeton bogneri*	MT226774
	Aroideae	*Syngonium podophyllum*	OK539580
	Aroideae	*Taccarum caudatum*	MN046895
	Aroideae	*Typhonium blumei*	MT161480
	Aroideae	*Xanthosoma sagittifolium*	MW628970
	Aroideae	*Zamioculcas zamiifolia*	MT226775
	Aroideae	*Zantedeschia rehmannii*	MH743154
	Aroideae	*Zomicarpella amazonica*	MT161483
	Lasioideae	*Lasia spinosa*	MT226772
	Lemnoideae	*Lemna minor*	DQ400350
	Lemnoideae	*Spirodela polyrhiza*	MN419335
	Lemnoideae	*Wolffia australiana*	MN912638
	Lemnoideae	*Wolffiella lingulata*	JN160604
	Monsteroideae	*Epipremnum amplissimum*	MN477424
	Monsteroideae	*Monstera adansonii*	MN046888
	Monsteroideae	*Spathiphyllum cannifolium*	MK372232
	Monsteroideae	*Stenospermation multiovulatum*	MN046893
	Orontioideae	*Orontium aquaticum*	MT226773
	Orontioideae	*Symplocarpus nipponicus*	MK341566
	Pothoideae	*Anthurium huixtlense*	MN996266
	Pothoideae	*Pothos chinensis*	PV938952
Tofieldiaceae		*Potamogeton perfoliatus*	KT899951
		*Sagittaria lichuanensis*	KT899952
		*Tofieldia ulleungensis*	OM640092
Acoraceae		*Acorus calamus*	MT755635

### 2.4 Phylogenetic Analysis Among Araceae Species

The available complete chloroplast genome of 46 aroid species representing 45 genera of Araceae and four Tofieldiaceae and Acoraceae species serving as outgroups were retrived from the GenBank database (Table 1). Then, 79 protein‐coding regions were extracted from the chloroplast genomes using Geneious Prime v2025.0.2 before being aligned using MUSCLE v5.3 with defautl settings (Edgar 2022). TrimAl v1.5.1 was used to remove gaps in the aligned sequences (Capella‐Gutiérrez et al. 2009). The trimmed dataset was subjected to jModeltest 2 to find the best substitution model which was identified as GTR + I + G (Darriba et al. 2012). For reconstructing phylogenetic relationships among aroid species, maximum likelihood (ML) and Bayesian inference (BI) methods were employed. For ML method, the IQ‐TREE 2 was used with settings of 1000 bootstraps alignments and substitution model of GTR + I + G (Minh et al. 2020). Similarly, for BI analysis, MrBayes v 3.2.7a was used with settingd of GTR + I + G substitution model, 3,000,000 generations, and 25% discarded samples (Ronquist et al. 2012). The phylogenetic trees were illustrated using Figtree v1.4.5 (https://github.com/rambaut/figtree/releases).

## 3 Results

### 3.1 Features of *H. perplexa* Chloroplast Genome

**FIGURE 2:**
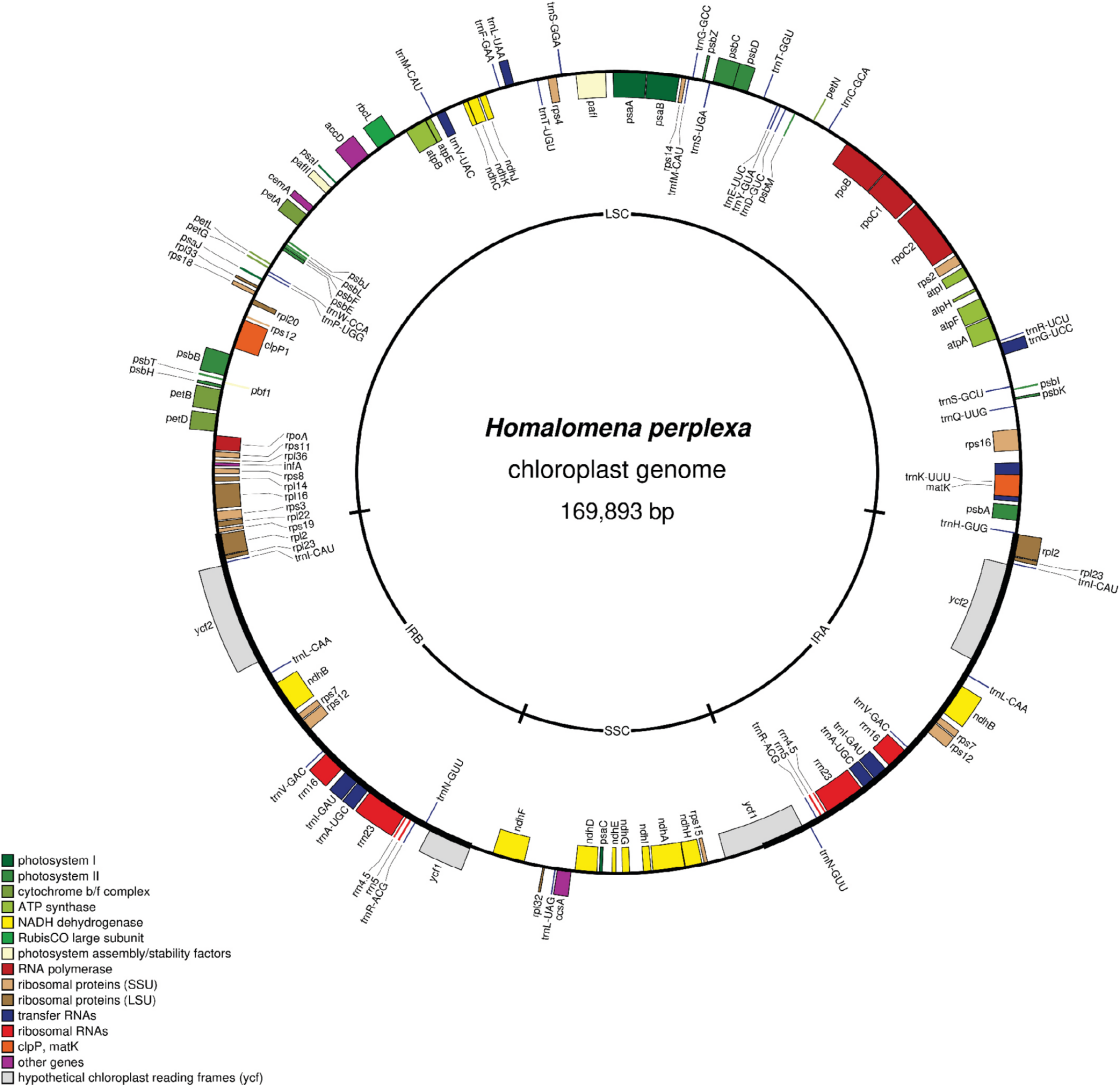
Chloroplast genome map of *Homalomena perplexa*. The inner and outer genes indicate the clockwise and counterclockwise translation directions, respectively. The inner circle indicates four regions of the chloroplast genome. IRA and IRB, Inverted repeat regions A and B, respectively; LSC, Large single‐copy region; SSC, Small single‐copy region.

**FIGURE 3:**
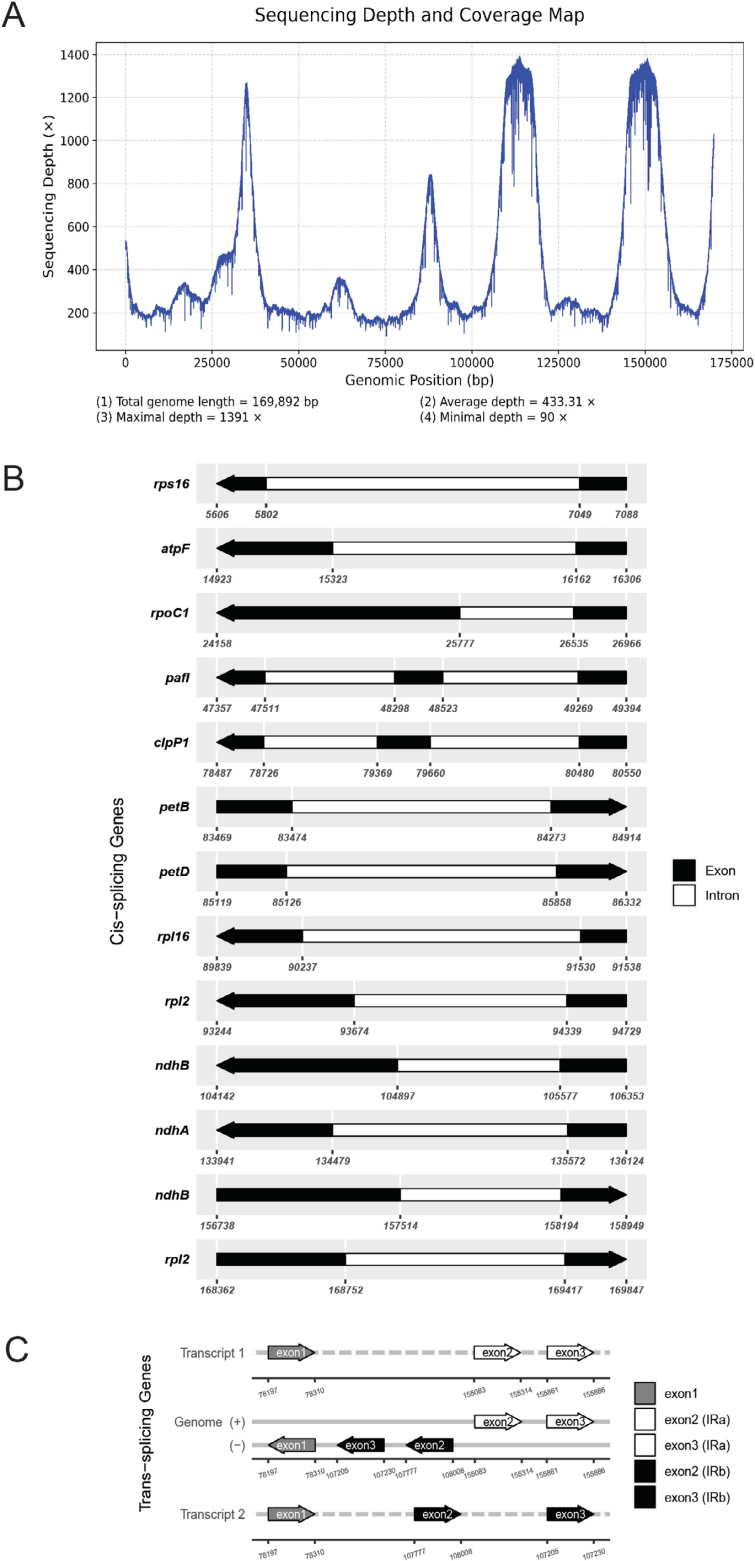
Coverage depth and trans‐splicing and cis‐splicing gene content in chloroplast genome of *Homalomena perplexa*. (A) Coverage depth of *H. perplexa* chloroplast genome. (B) Cis‐splicing genes of *H. perplexa* chloroplast genome. C. Trans‐splicing gene of *H. perplexa* chloroplast genome.

The complete chloroplast genome of *H. perplexa* (with average coverage depth of 433×) was 169,893 bp in length and had 35.4% GC content (Figures 2 and 3A). This circular genome consisted of a large‐single copy (LSC, 93,197 bp in length and 33.4% GC content), a small‐single copy (SSC, 20,014 bp in length and 27.3% GC content), and two identical inverted repeat regions (IR, 28,341 bp in length and 41.4% GC content each) (Figure 2). In Addition, there were 113 unique coding regions in this quadripartite genome, including 79 protein‐coding genes, 30 transfer RNA genes, and four ribosomal RNA genes (Figure 2, Table 2). Among coding regions, *pafI* and *clpP1* genes had two introns whereas *rps16*, *atpF*, *rpoC1*, *petB*, *petD*, *rpl16*, *rpl2*, *ndhB*, *ndhA, trnK_UUU, trnI_GAU*, *trnA_UGC*, *trnG_UCC*, *trnL_UAA*, and *trnV_UAC* each had one intron (Figure 3B). The *rps12* gene was transpliced, of which the exon 1 was in the LSC region whereas the exon 2 and exon 3 were in the IR regions (Figure 3C). Due to the presence of IR regions, *rpl2*, *rpl23*, *trnI_CAU*, *ycf2*, *trnL_CAA*, *ndhB*, *rps7*, *rps12*, *trnV_GAC*, *rrn16*, *trnI_GAU*, *trnA_UGC*, *rrn23*, *rrn4.5*, *rrn5*, *trnR_ACG*, *trnN_GUU*, and *ycf1* were duplicated in the IR region, but *ycf1* was present as an incomplete duplication (Figure 2, Table 2).

**TABLE 2:** Gene composition of *Homalomena perplexa* chloroplast genome.

Groups of genes	Name of genes
Ribosomal RNAs	*rrn4.5* ^a^, *rrn5* ^a^, *rrn16* ^a^, *rrn23* ^a^
Transfer RNAs	*trnA_UGC* ^a^, ^b^, *trnC_GCA*, *trnD_GUC*, *trnE_UUC*, *trnF_GAA*, *trnG_UCC* ^b^, *trnG_GCC*, *trnH_GUG*, *trnI_GAU* ^a^, ^b^, *trnK_UUU* ^b^, *trnL_CAA* ^a^, *trnL_UAA* ^b^, *trnL_UAG*, *trnfM_CAU*, *trnI_CAU* ^a^, *trnM_CAU*, *trnN_GUU* ^a^, *trnP_UGG*, *trnQ_UUG*, *trnR_ACG* ^a^, *trnR_UCU*, *trnS_GCU*, *trnS_GGA*, *trnS_UGA*, *trnT_GGU*, *trnT_UGU*, *trnV_GAC* ^a^, *trnV_UAC* ^b^, *trnW_CCA*, *trnY_GUA*
Large units of ribosome	*rpl2* ^a^, ^b^, *rpl14*, *rpl16* ^b^, *rpl20*, *rpl22*, *rpl23* ^a^, *rpl32*, *rpl33*, *rpl36*
Small units of ribosome	*rps2*, *rps3*, *rps4*, *rps7* ^a^, *rps8*, *rps11*, *rps12* ^a^, *rps14*, *rps15*, *rps16* ^b^, *rps18*, *rps19*
RNA polymerase	*rpoA*, *rpoB*, *rpoC1* ^b^, *rpoC2*
Translational initiation factor	*infA*
Subunit of photosystem I	*psaA*, *psaB*, *psaC*, *psaI*, *psaJ*, *pafI* ^c^, *pafII*
Subunit of photosystem II	*psbA*, *psbB*, *psbC*, *psbD*, *psbE*, *psbF*, *psbH*, *psbI*, *psbJ*, *psbK*, *psbL*, *pbfI*, *psbM*, *psbT*, *psbZ*
Subunit of cytochrome	*petA*, *petB* ^b^, *petD* ^b^, *petG*, *petL*, *petN*
Subunit of ATP synthases	*atpA*, *atpB*, *atpE*, *atpF* ^b^, *atpH*, *atpI*
Large unit of Rubisco	*rbcL*
Subunit of NADH dehydrogenase	*ndhA* ^b^, *ndhB* ^a^, ^b^, *ndhC*, *ndhD*, *ndhE*, *ndhF*, *ndhG*, *ndhH*, *ndhI*, *ndhJ*, *ndhK*
Maturase	*matK*
Envelope membrane protein	*cemA*
Subunit of acetyl‐CoA	*accD*
C‐type cytochrome synthesis gene	*ccsA*
ATP‐dependent protease subunit P	*clpP1* ^c^
Hypothetical proteins and conserved reading frames	*ycf1* ^a^, *ycf2* ^a^

^a^ Duplicated gene in IR region.

^b^ Genes containing single intron.

^c^ Genes containing two introns.

In the *H. perplexa* chloroplast genome, there were 81 SSRs composed of 63 mononucleotide repeats, 16 dinucleotide repeats, and two trinucleotide repeats (Table 3). All these repeats were made up of A and T nucleotides. Similarly, *H. occulta* chloroplast genome possessed 79 SSRs, including 63 mononucleotide repeats, 12 dinucleotide repeats, and four trinucleotide repeats. Most of the SSRs in the *H. occulta* chloroplast genome contained A and T nucleotides, except one trinucleotide repeat made up of the TCC unit. Among identified SSRs, there were 62 shared repeats between two *Homalomena* chloroplast genomes, which included 52 mononucleotide repeats, eight dinucleotide repeats, and two trinucleotide repeats. However, *H. occulata* had four and two unique dinucleotide and trinucleotide repeats, respectively. Meanwhile, *H. perplexa* had eight unique dinucleotide repeats. There were no records of tetranucleotide, pentanucleotide, and hexanucleotide repeats in the two *Homalomena* chloroplast genomes examined. In addition, the length of mononucleotide repeats ranged from 10 to 16 bp in *H. perplexa* and from 10 to 18 bp in *H. occulta*. Similarly, *H. occulta* had 12–18 bp dinucleotide repeats, whereas *H. perplexa* contained 12–14 bp dinucleotide repeats. The length of trinucleotide repeats (15 bp) was identical in both *Homalomena* species (Table 3).

**TABLE 3:** Features of SSRs in chloroplast genomes of two examined *Homalomena* species.

*Homalomena perplexa* PX569150				*Homalomena occulta* MW145396			
Type	Repeat Unit/length (bp)	Location		Type	Repeat Unit/length (bp)	Location	
		Start	End			Start	End
Dinucleotide	TA/12	231	243	Dinucleotide	TA/12	231	243
Mononucleotide	A/10	2093	2102	Mononucleotide	A/11	2132	2142
Mononucleotide	T/10	5920	5929	Mononucleotide	T/11	5959	5969
Mononucleotide	A/10	6078	6087	Mononucleotide	A/10	6110	6119
Mononucleotide	A/14	7407	7420	Mononucleotide	A/14	7413	7426
Mononucleotide	A/10	7430	7439	Mononucleotide	A/10	7436	7445
Mononucleotide	T/10	10,980	10,989	Mononucleotide	T/10	10,890	10,899
Mononucleotide	A/10	11,021	11,030	Mononucleotide	A/11	10,931	10,941
Mononucleotide	T/11	11,093	11,103	Mononucleotide	T/10	11,004	11,013
Mononucleotide	A/11	11,482	11,492	Mononucleotide	A/11	11,343	11,353
Mononucleotide	A/11	13,083	13,093	Mononucleotide	A/14	12,910	12,923
Mononucleotide	T/11	13,201	13,211	Mononucleotide	T/11	13,031	13,041
Dinucleotide	TA/12	16,073	16,084	Dinucleotide	TA/12	15,901	15,912
Mononucleotide	A/10	16,101	16,110	Mononucleotide	A/12	15,940	15,951
Mononucleotide	A/14	17,091	17,104	Mononucleotide	A/15	16,931	16,945
Mononucleotide	T/11	19,668	19,678	Mononucleotide	T/11	19,507	19,517
Mononucleotide	T/11	21,861	21,871	Mononucleotide	T/11	21,705	21,715
Mononucleotide	T/12	25,883	25,894	Mononucleotide	T/14	25,722	25,735
Mononucleotide	T/10	29,568	29,577	Mononucleotide	T/10	29,409	29,418
Mononucleotide	T/11	30,718	30,728	Mononucleotide	T/13	30,554	30,566
Mononucleotide	A/10	34,782	34,791	Mononucleotide	A/12	34,605	34,616
Dinucleotide	TA/12	41,070	41,082	Dinucleotide	TA/12	40,915	40,927
Mononucleotide	T/12	41,094	41,105	Mononucleotide	T/14	40,937	40,950
Mononucleotide	A/10	41,684	41,693	Mononucleotide	A/10	41,533	41,542
Mononucleotide	A/10	41,702	41,711	Mononucleotide	A/11	41,551	41,561
Mononucleotide	T/10	47,293	47,302	Mononucleotide	T/10	47,143	47,152
Mononucleotide	A/12	49,236	49,247	Mononucleotide	A/12	49,099	49,110
Mononucleotide	A/10	49,498	49,507	Mononucleotide	A/11	49,361	49,371
Mononucleotide	A/10	50,504	50,513	Mononucleotide	A/10	50,373	50,382
Dinucleotide	TA/12	52,853	52,864	Dinucleotide	TA/18	52,465	52,482
Dinucleotide	TA/12	55,009	55,020	Dinucleotide	TA/12	54,633	54,644
Mononucleotide	T/11	59,495	59,505	Mononucleotide	T/12	59,128	59,139
Mononucleotide	A/12	61,701	61,712	Mononucleotide	A/16	61,335	61,350
Mononucleotide	T/10	61,993	62,002	Mononucleotide	T/10	61,629	61,638
Mononucleotide	T/10	69,091	69,100	Mononucleotide	T/11	68,736	68,746
Mononucleotide	A/10	69,297	69,306	Mononucleotide	A/10	68,943	68,952
Mononucleotide	A/13	69,315	69,327	Mononucleotide	A/11	68,961	68,971
Dinucleotide	AT/12	71,962	71,974	Dinucleotide	AT/12	71,612	71,624
Mononucleotide	T/11	75,308	75,318	Mononucleotide	T/12	74,957	74,968
Mononucleotide	T/11	79,334	79,344	Mononucleotide	T/13	79,006	79,018
Mononucleotide	T/11	82,567	82,576	Mononucleotide	T/10	82,242	82,251
Mononucleotide	A/13	83,381	83,393	Mononucleotide	A/14	83,056	83,069
Mononucleotide	T/10	86,460	86,469	Mononucleotide	T/11	86,136	86,146
Mononucleotide	A/11	86,506	86,516	Mononucleotide	A/11	86,183	86,193
Mononucleotide	A/11	88,327	88,337	Mononucleotide	A/10	88,004	88,013
Mononucleotide	T/11	90,857	90,867	Mononucleotide	T/13	90,520	90,532
Mononucleotide	T/12	91,338	91,349	Mononucleotide	T/11	91,003	91,013
Dinucleotide	TA/12	103,544	103,556	Dinucleotide	TA/12	103,208	103,220
Mononucleotide	T/12	106,409	106,420	Mononucleotide	T/12	106,073	106,084
Mononucleotide	T/10	112,187	112,196	Mononucleotide	T/10	111,848	111,857
Mononucleotide	A/16	117,223	117,238	Mononucleotide	A/18	116,891	116,908
Trinucleotide	TAA/15	118,347	118,361	Trinucleotide	TAA/15	118,012	118,026
Mononucleotide	T/10	126,208	126,217	Mononucleotide	T/11	122,104	122,114
Mononucleotide	A/11	128,528	128,538	Mononucleotide	A/11	124,413	124,423
Mononucleotide	T/11	131,797	131,807	Mononucleotide	T/11	127,702	127,712
Mononucleotide	T/10	132,734	132,743	Mononucleotide	T/10	128,629	128,638
Mononucleotide	T/11	137,709	137,719	Mononucleotide	T/11	133,410	133,420
Trinucleotide	TTA/15	144,730	144,744	Trinucleotide	TTA/15	140,234	140,248
Mononucleotide	T/16	145,853	145,868	Mononucleotide	T/18	141,352	141,369
Mononucleotide	A/10	150,895	150,904	Mononucleotide	A/10	146,403	146,412
Mononucleotide	A/12	156,671	156,682	Mononucleotide	A/12	152,176	152,187
Dinucleotide	AT/12	159,535	159,547	Dinucleotide	AT/12	155,040	155,052
**Mononucleotide**	**T/10**	**563**	**572**	**Dinucleotide**	**TA/12**	**274**	**286**
**Dinucleotide**	**TA/14**	**10,262**	**10,276**	**Mononucleotide**	**A/11**	**5310**	**5320**
**Mononucleotide**	**T/11**	**18,836**	**18,846**	**Mononucleotide**	**A/12**	**9863**	**9874**
**Mononucleotide**	**A/10**	**33,638**	**33,647**	**Mononucleotide**	**A/14**	**36,803**	**36,816**
**Mononucleotide**	**A/10**	**40,979**	**40,988**	**Dinucleotide**	**AT/12**	**54,557**	**54,568**
**Mononucleotide**	**A/11**	**54,021**	**54,031**	**Mononucleotide**	**A/10**	**57,055**	**57,064**
**Mononucleotide**	**T/12**	**54,851**	**54,862**	**Mononucleotide**	**T/10**	**63,847**	**63,856**
**Dinucleotide**	**AT/12**	**54,937**	**54,948**	**Mononucleotide**	**T/13**	**66,134**	**66,146**
**Mononucleotide**	**T/10**	**57,337**	**57,346**	**Dinucleotide**	**TA/12**	**71,007**	**71,018**
**Mononucleotide**	**T/10**	**89,795**	**89,804**	**Mononucleotide**	**T/10**	**73,869**	**73,878**
**Mononucleotide**	**T/11**	**90,806**	**90,816**	**Mononucleotide**	**A/11**	**79,574**	**79,584**
**Dinucleotide**	**TA/12**	**122,335**	**122,346**	**Mononucleotide**	**T/17**	**88,906**	**88,922**
**Dinucleotide**	**TA/14**	**122,351**	**122,364**	**Mononucleotide**	**A/10**	**128,817**	**128,826**
**Dinucleotide**	**AT/12**	**122,647**	**122,658**	**Mononucleotide**	**A/10**	**133,086**	**133,095**
**Dinucleotide**	**TA/14**	**122,659**	**122,672**	**Trinucleotide**	**TAT/15**	**133,910**	**133,924**
**Dinucleotide**	**AT/14**	**122,693**	**122,707**	**Dinucleotide**	**AT/14**	**134,299**	**134,312**
**Mononucleotide**	**A/10**	**122,936**	**122,945**	**Trinucleotide**	**TCC/15**	**137,686**	**137,702**
**Mononucleotide**	**T/11**	**138,228**	**138,238**				
**Dinucleotide**	**TA/12**	**138,469**	**138,481**				

*Note:* Bold words and numbers indicate specific SSR in each *Homalomena* chloroplast genome.

**FIGURE 4:**
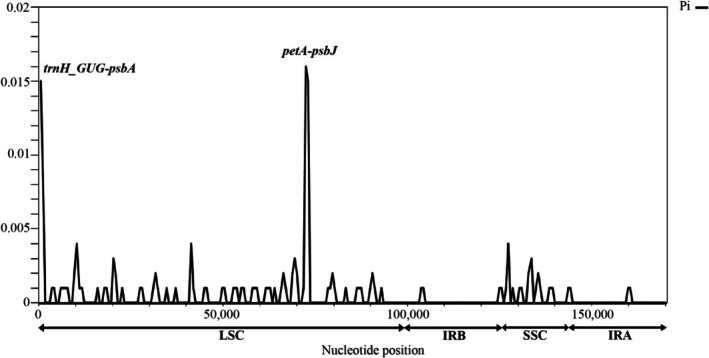
Nucleotide diversity among two *Homalomena* chloroplast genomes. Nucleotide positions are shown on the x‐axis and Pi values are shown on the y‐axis. IRA and IRB, Inverted repeat regions A and B, respectively; LSC, Large‐single copy region; SSC, Small‐single copy region.

Analysis of long repeats revealed the presence of forward and palindromic repeats in the chloroplast genomes of *H. occulta* and *H. perplexa* (Table 4). There were 23 forward repeats (ranging from 20 to 34 bp) and 13 palindromic repeats (ranging from 20 to 29 bp) in *H. occulta*. Meanwhile, *H. perplexa* had 26 forward repeats (ranging from 20 to 38 bp) and 21 palindromic repeats (ranging from 20 to 23 bp). Between *H. occulta* and *H. perplexa* there were 11 shared forward repeats and 10 shared palindromic repeats, which ranged from 20 bp to 34 bp. In addition, there were 26 and 15 unique long repeats in *H. perplexa* and *H. occulta* chloroplast genomes, respectively (Table 4). Notably, there were no reverse and complement repeats in the two examined chloroplast genomes of *Homalomena*. Comparative analysis between *H. occulta* and *H. perplexa* chloroplast genomes revealed a high similarity (Figure 4). Only two regions (including *trnH_GUG‐psbA* and *petA‐psbJ*) had Pi values over 0.015. In contrast, most of the IRA and IRB regions had Pi values equal to 0. Other regions possessed Pi values under 0.005.

**TABLE 4:** Features of long repeats in chloroplast genomes of two examined *Homalomena* species.

Type/Length (bp)	Sequence	Location in *Homalomena occulta* MW145396		Location in *Homalomena perplexa PX569150*	
		Start	End	Start	End
Forward/34	TTTGTCTAAGTCACTTCGTTTCTTTTTGTCCAAG	97,625	97,658	97,961	97,994
		97,670	97,703	98,006	98,039
Forward/34	GATATCGATATTGATGATAGTGACGATATTGATA	100,086	100,119	100,422	100,455
		100,134	100,167	100,470	100,503
Forward/32	AGACTACACACTACTAATTATATTATATAATA	133,753	133,784	138,052	138,083
		133,791	133,822	138,090	138,121
Forward/29	ATTTATTTATTATATATTTATTTATTTTT	177	205	177	205
		31,019	31,047	31,164	31,192
Palindromic/29	AAAAGTAAGAACTCAGCAGGACCGTACCC	71,917	71,945	72,267	72,295
		71,967	71,995	72,317	72,345
Palindromic/26	TATTTTAATTTTATTTATTATTATAT	210	235	210	235
		51,599	51,624	51,734	51,759
Forward/26	TTTATTTAATTTATTTATTTAATATT	30,976	31,001	31,121	31,146
		52,549	52,574	52,915	52,940
Forward/25	GGAGAGAGAGGGATTCGAACCCTCG	9926	9950	9936	9960
		40,130	40,154	40,284	40,308
Forward/25	TATGTATTTGAAATACAATTGTTAT	10,755	10,779	10,845	10,869
		10,789	10,813	10,879	10,903
Palindromic/25	AAGTTTTTTTGAGAACCATTTGACT	124,452	124,476	128,567	128,591
		124,482	124,506	128,597	128,621
Palindromic/24	AGAGAGGGATTCGAACCCTCGGTA	9930	9953	9940	9963
		50,214	50,237	50,345	50,368
Palindromic/23	GAAGTAATGAACCTCCCAATATG	82,522	82,544	82,847	82,869
		82,546	82,568	82,871	82,893
Palindromic/24	ATTTTTCTATTTTTTCTTTTAAT	122,796	122,818	126,903	126,926
		122,828	122,850	126,934	126,957
Forward/22	GATGCGGGTTCGATTCCCGCTA	12,752	12,773	12,925	12,946
		41,368	41,389	41,519	41,540
Palindromic/21	AATAATATAATAATAAATAAA	51,595	51,615	51,730	51,750
		126,788	126,808	130,905	130,925
Forward/21	TAATATTAATATAATGTAATA	66,234	66,254	66,600	66,620
		66,250	66,270	66,616	66,636
Palindromic/21	ATTATATATTATATAATATAA	75,959	75,979	76,309	76,329
		133,808	133,828	138,107	138,127
Forward/21	TATTATAATTTAAATTTAATT	118,915	118,935	123,023	123,043
		128,759	128,779	133,067	133,087
Forward/20	ATATTTTATTATATTTCATT	31,002	31,021	31,147	31,166
		126,765	126,784	130,882	130,901
Palindromic/20	AACCACTCGGCCATCTCTCC	40,202	40,221	40,356	40,375
		50,156	50,175	50,287	50,306
Palindromic/20	TATATATATTATATAAAATAA	10,250	10,270	10,283	10,302
		86,145	86,165	86,468	86,487
Palindromic/20	TTTTTTTTTTATTTTATATA	86,137	86,156	n/a	n/a
		128,807	128,826	n/a	n/a
Forward/20	AATATCAATATCCAAAATAA	103,230	103,249	n/a	n/a
		103,248	103,267	n/a	n/a
Forward/20	TATATATATTTTTATATATT	31,059	31,078	n/a	n/a
		40,919	40,938	n/a	n/a
Forward/20	TATATATATATTTAATTATT	233	252	n/a	n/a
		255	274	n/a	n/a
Forward/21	ATAATATAACATATATAAATA	75,971	75,991	n/a	n/a
		75,992	76,012	n/a	n/a
Forward/21	TAAAAAAAAAAAAGAAATAAA	34,604	34,624	n/a	n/a
		49,098	49,118	n/a	n/a
Forward/22	ATATATATATAATATATATATA	15,902	15,923	n/a	n/a
		71,601	71,622	n/a	n/a
Palindromic/22	TATTATATTTATTATATTTATA	41,048	41,069	n/a	n/a
		90,276	90,297	n/a	n/a
Forward/21	ATTATTATATATATATATTTA	226	246	n/a	n/a
		269	289	n/a	n/a
Forward/21	TATATTTAAATTATATATATT	10,205	10,225	n/a	n/a
		10,220	10,240	n/a	n/a
Forward/23	ATTATATTATTATATCATACTTA	126,798	126,820	n/a	n/a
		126,831	126,853	n/a	n/a
Forward/24	TTATTTATATATTATTTATATATT	128,536	128,559	n/a	n/a
		128,547	128,570	n/a	n/a
Palindromic/23	TATTATATTATAATATAAATATA	10,353	10,375	n/a	n/a
		66,857	66,879	n/a	n/a
Forward/26	TATATATTTATAATTATATTTTATAT	35,243	35,268	n/a	n/a
		35,266	35,291	n/a	n/a
Forward/25	ATTTAAATAATATTTAATTTAAATA	6025	6049	n/a	n/a
		6041	6065	n/a	n/a
Forward/20	TAAATATATATTATAATATA	n/a	n/a	10,438	10,457
		n/a	n/a	10,489	10,508
Palindromic/20	TATATATATATTTTTTTTTT	n/a	n/a	41,084	41,103
		n/a	n/a	49,498	49,517
Palindromic/20	TTTTTCAAAAAAAAAAAAGA	n/a	n/a	61,695	61,714
		n/a	n/a	91,336	91,355
Forward/20	AATTATAAATATAATAAATA	n/a	n/a	90,601	90,620
		n/a	n/a	90,664	90,683
Palindromic/20	CCTTTTTACGTCCCCATGTC	n/a	n/a	111,600	111,619
		n/a	n/a	111,633	111,652
Palindromic/20	TATTAATATATAAAATAAAA	n/a	n/a	133,109	133,128
		n/a	n/a	138,653	138,672
Forward/20	ATATTTCATTTATTTATTAT	n/a	n/a	31,157	31,176
		n/a	n/a	132,594	132,613
Palindromic/20	ATAAAATAAAAAAAAAAGAA	n/a	n/a	34,775	34,794
		n/a	n/a	86,457	86,476
Palindromic/20	ATAATATATATTTATATTAT	n/a	n/a	262	281
		n/a	n/a	122,291	122,310
Forward/21	TATAACAATATATAATTTATA	n/a	n/a	122,069	122,089
		n/a	n/a	122,086	122,106
Forward/21	TATATATATATATAATATAAA	n/a	n/a	122,659	122,679
		n/a	n/a	122,694	122,714
Forward/21	ATTTATTTATTATTTATATTT	n/a	n/a	66,471	66,491
		n/a	n/a	132,601	132,621
Palindromic/22	TATATATATATAATATATATAT	n/a	n/a	55,009	55,030
		n/a	n/a	122,649	122,670
Forward/22	TATTTATAATTTTATAATATAT	n/a	n/a	138,499	138,520
		n/a	n/a	138,520	138,541
Palindromic/21	ATATATTATATAATATATATT	n/a	n/a	10,271	10,291
		n/a	n/a	122,299	122,319
Palindromic/21	AAATATAAATATAATTATAAA	n/a	n/a	11,576	11,596
		n/a	n/a	41,033	41,053
Forward/22	AACTATGACTATAAATAATAAA	n/a	n/a	6110	6131
		n/a	n/a	6130	6151
Forward/22	ACCTTTTTTAATAATAATATTA	n/a	n/a	10,094	10,115
		n/a	n/a	10,131	10,152
Forward/22	ATATAAAATAAATATTAAAATA	n/a	n/a	11,506	11,527
		n/a	n/a	66,544	66,565
Palindromic/23	ATAAATATAATTAAATATAATAT	n/a	n/a	11,432	11,454
		n/a	n/a	52,600	52,622
Palindromic/23	ATATATATATAATATATATATAT	n/a	n/a	71,951	71,973
		n/a	n/a	122,647	122,669
Forward/25	ATATTATAATATATAAAATATAAAT	n/a	n/a	10,458	10,482
		n/a	n/a	10,496	10,520
Forward/27	ATTATTATAATAATTATAATAAAATAT	n/a	n/a	122,584	122,610
		n/a	n/a	122,609	122,635
Forward/26	ATATAAATATAATTATAAATATAATA	n/a	n/a	11,578	11,603
		n/a	n/a	90,591	90,616
Forward/38	ATATATTAAATATTATATTATTATAATTATATATATTA	n/a	n/a	138,549	138,586
		n/a	n/a	138,579	138,616
Forward/21	ATTTATATATATATATTTATA	n/a	n/a	41,067	41,087
		n/a	n/a	138,466	138,486

Abbreviation: n/a, no data.

### 3.2 Variations of Junctions Among the LSC, SSC and IR Regions in Araceae Chloroplast Genomes

**FIGURE 5:**
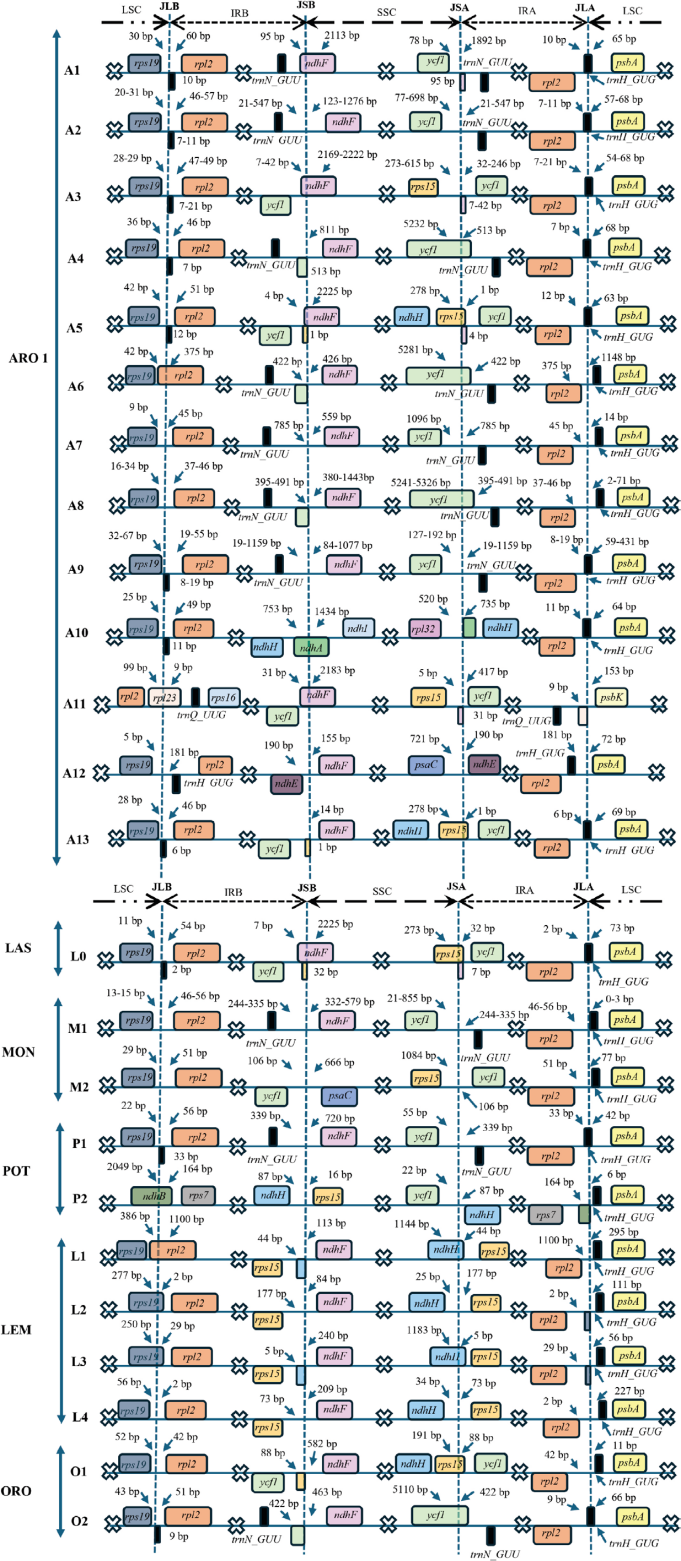
Junctions among LSC, SSC, and IR regions of Araceae species. JLB: Junction between LSC and IRB regions. JSB: Junction between SSC and IRB regions. JSA: Junction between SSC and IRA regions. JLA: Junction between LSC and IRA regions. The vertical dashed lines indicate the junction sites. The lines represent the sequences and regions and do not show exact length‐based ratios in the chloroplast genomes. Multiplication signs indicate other contents in the chloroplast genomes.ARO, Aroideae; LAS, Lasioideae; LEM, Lemnoideae; MON, Monsteroideae; ORO, Orontioideae; POT, Pothoideae.

Comparative genome structure analysis among 46 examined Araceae species revealed different junctions among LSC, SSC, and IR regions (Figure 5). In Aroideae, there were 13 junction variations inferred from 32 examined genera, whereas two variations were identified in four genera of Monsteroideae. In Lemnoideae, Orontioideae, Pothoideae, and Lasioideae, the number of junction variations was identical to the number of examined genera, which were 4,2,2, and 1, respectively. Although various junction variations were found, the gene content at the boundaries was quite similar. Specifically, the junction between the LSC and IRB regions (JLB) located in the intergenic spacer (IGS) between the *rps19* and *rpl22* genes. In some cases, the JLB moved to coding regions of *rpl2* (i.e., L1 of Lemnoideae), *rps19* (i.e., L2 and L3 of Lemnoideae), *rpl23* (i.e., A11 of Aroideae), and *ndhB* (i.e., P2 of Pothoideae). In the case of the junction between the LSC and IRA regions (JLA), there was a shift of *trnH_GUG* from the LSC region to the IRA region until the whole *trnH_GUG* was located in the IRA region (i.e., A11 of Aroideae). Notably, due to the expansion of the IR region to the LSC region in *Anchomanes hookeri* (A11 of Aroideae), the JSA contained partial *ndhF* and near *psbK*. Similar to JLA and JLB, the junctions among SSC, IRA, and IRB regions (JSA and JSB) exhibited a movement to include *ycf1* and other genes in the SSC region. For example, the JSA located in the IGS between *ycf1* and *trnN_GUU* (i.e., A2, A7, and A9 of Aroideae) and then found in the coding region of *ycf1* (i.e., A4, A6, and A8 of Aroideae). Continuously, the JSA was in the IGS between *ycf1* and *rps15* (i.e., A3 and A11 of Aroideae) and located within *ndhA* (i.e., A10 of Aroideae) before being identified in the coding region of *rps15* (i.e., A5 and A13 of Aroideae). Notably, in A12 variation (belonging to *Zantedeschia rehmannii*), the JSA was in the IGS between *psaC* and *ndhE*. In the JSB, there were also records of *ndhF* overlap in A1, A3, A5, and A11 variations, which ranged from 4 to 95 bp. A similar trend of JSA and JSB was found in the chloroplast genomes of other Araceae subfamilies (Figure 5).

### 3.3 Phylogenetic Relationships Among Examined Areaceae Species

**FIGURE 6:**
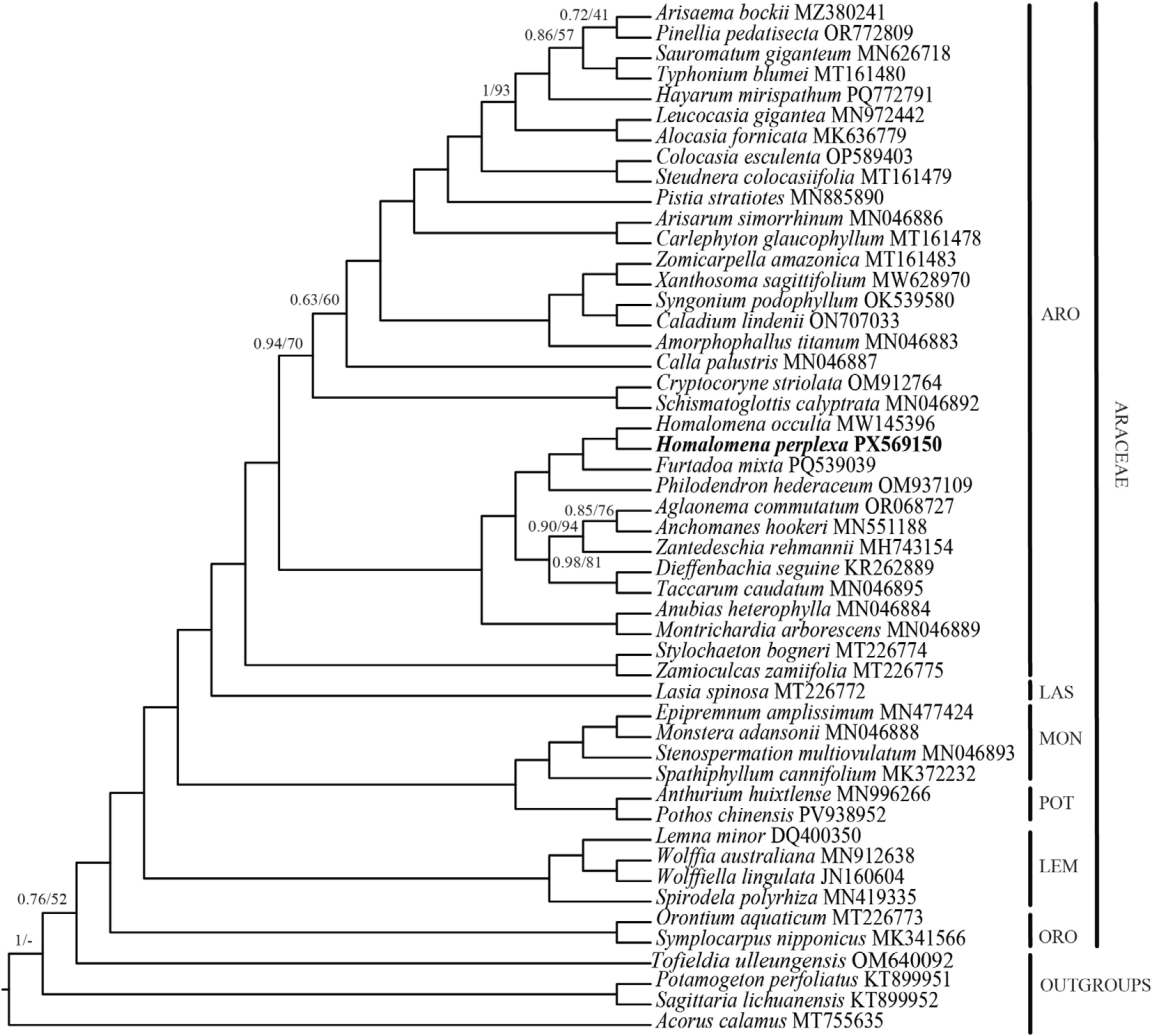
Phylogenetic tree in cladogram format of 46 Araceae species inferred from 79 plastid protein‐coding genes using maximum likelihood and Bayesian inference methods. Only posterior probability ≤ 1 and bootstrap values ≤ 100 were shown at the nodes. ARO, Aroideae; LAS, Lasioideae; LEM, Lemnoideae; MON, Monsteroideae; ORO, Orontioideae; POT, Pothoideae.

Phylogenetic analysis inferred from 79 protein‐coding genes using ML and BI methods resulted in identical topology of phylogenetic trees (Figure 6). The monophyly of Araceae subfamilies was highly supported with posterior probability = 1 and bootstrap value = 100. Within Aroideae, there were two distinct clades with strong support values. However, phylogenetic relationships among *Ariseama bockii, Pinellia pedatisecta, Aglaonema commutatum, Anchomanes hookeri, Calla palustris*, and related species in Aroideae exhibited low and moderate support values. *Homalomena perplexa* formed a clade with *H. occulta* which was sister to *Furtadoa mixta* with high support values (PP = 1 and BS = 100).

## 4 Discussion

The complete chloroplast genome of *H. perplexa* revealed a conserved pattern regarding gene content and genome structure, which was found in other angiosperms (Dobrogojski et al. 2020). Within Araceae, the quadripartite structure and a unique profile of 79 protein‐coding genes, 30 tRNAs, and four rRNAs were commonly found in the published chloroplast genomes (Abdullah, Henriquez, Mehmood, Shahzadi, et al. 2020; Henriquez et al. 2020; Abdullah et al. 2021; Zhang et al. 2021; Li et al. 2022, 2025). However, gene loss has been recorded in the chloroplast genomes of *Amorphophallus* species (Liu et al. 2019; Li et al. 2024). In particular, the *ycf1* gene was absent in *Amorphophallus bulbifer*, whereas *Amorphophallus konjac* did not have *psbE* and *trnG_GCC*. In addition, *trnL‐CAA* was lost in *Amorphophallus muelleri* and *A. bulbifer* and only *A. muelleri* had the *accD* gene. The *infA* gene was only detected in *Amorphophallus titanum* (Li et al. 2024). Similarly, within Araceae, the loss of *infA* was found in *Colocasia*, *Dieffenbachia*, *Spirodela*, *Lemna*, *Wolffiella*, and *Wolffia* (Choi et al. 2017). These results indicate a dynamic pattern of gene loss in the *Amorphophallus* genus and other Araceae genera. Although gene loss was not observed in the current study, many unexplored *Homalomena* chloroplast genomes may reveal different genomic events. Furthermore, the impacts of gene loss in Araceae require further investigations.

Although the chloroplast genomes of *H. perplexa* and *H. occulta* showed a high similarity in terms of shared repeats (62 SSRs and 21 long repeats), they still had unique repeat sequences. Previously, dynamic repeat contents have been characterized among Araceae chloroplast genomes; however, the shared and unique repeats have not been assessed (Choi et al. 2017; Liu et al. 2019; Abdullah, Henriquez, Mehmood, Carlsen, et al. 2020). The lack of information about shared and unique repeats might be caused by the deficiency of chloroplast genomes among the examined plant genera and families. Due to a dramatic increase in chloroplast genome data recently, the shared and unique repeats in different genera and families should be analyzed to provide a new aspect of genomic evolution in land plants. In chloroplast genomes, repeats are useful sources for exploring population genetics, gene flow, and genetic diversity (George et al. 2015; Feng et al. 2023; Huy et al. 2025; Moosavi et al. 2025). The shared and unique repeats in *Homalomena* chloroplast genomes are helpful in tracing the genomic evolution within the genus and among its species.

In addition to gene loss and conservation of quadripartite structure, Araceae chloroplast genomes possessed high variations in the junctions among LSC, SSC, and IR regions. A previous study indicated that the LSC/SSC/IR junctions were diverse according to plant groups (i.e., angiosperms, gymnosperms, ferns, lycophytes, hornworts, mosses, liverworts, and green algae) (Zhu et al. 2016). In the current study, the LSC/SSC/IR junctions of Araceae exhibited the features of angiosperms, which expanded to include *rps19* genes. Notably, the junction variation occurred in three directions, including the expansion from the IR regions to LSC region, from the IRA region to SSC and from the IRB region to the SSC region. Consequently, different junction variations were observed in Aroideae (13 variations), Pothoideae (two variations), Monsteroideae (two variations), and Lemnoideae (four variations). Although Aroideae has the largest number of variations, it does not indicate that Aroideae chloroplast genomes are more diverse than other subfamilies. The reason for the outstanding Aroideae is the availability of chloroplast genomes (belonging to 32 genera) compared to other subfamilies (fewer than five genera). Therefore, more aroid genera and species should be included to examine the junction variations. Within the Araceae genus, junction variations were also found in 13 *Amorphophallus* chloropalst genomes (Li et al. 2024). The two chloroplast genomes of *Homalomena* in the current study did not show a significant variation; however, the unexplored *Homalomena* species may contain more variations and genomic events that require further investigation.

Previously, a phylogenetic study of 102 aroid genera based on *rbcL, matK*, partial *trnK* intron, partial tRNA‐Leu gene, *trnL–trnF* spacer, and partial tRNA‐Phe gene revealed the backbone phylogeny of Araceae (Cusimano et al. 2011). Specifically, Gymnostachydoideae and Orontoideae formed a basal clade of Araceae. Pothoideae was close to Monsteroideae. Meanwhile, Zamioculcadoideae was embedded in Aroideae. In 2014, 70 plastid protein‐coding genes in 37 genera of Araceae were employed to reconstruct the phylogenetic relationships of which the infrafamilial relationships were similar to the previous result, except for the absence of Gymnostachydoideae (Henriquez et al. 2014). Recently, the plastid data set covering 79 Araceae genera and Angiosperms 353 nuclear data representing 111 Araceae genera were used to confirm the infrafamilial classification of Araceae (Haigh et al. 2023). The results from nuclear and plastid data showed similar relationships among subfamilies, except for the absence of Gymnostachydoideae in the plastid dataset. Consequently, they suggested the expansion of Aroideae to include Zamioculcadoideae and proposed a seven‐subfamily system of Araceae, of which Gymnostachydoideae and Orontoideae was basal clade of Araceae. In the current study, although the phylogenetic analysis did not reveal any alterations in infrafamilial relationships compared to previous studies, the data of Gymnostachydoideae were still absent. Therefore, the complete chloroplast genome of *Gymnostachys anceps*, a monotypic species of Gymnostachydoideae, is required for fulfilling the phyogenetic relationships of Araceae based on chloroplast genome data.

## Author Contributions

**Nhat Nam Nguyen:** conceptualization (equal), formal analysis (equal), methodology (equal), supervision (equal), writing – original draft (equal). **Ngoc Trai Nguyen:** formal analysis (equal), writing – original draft (equal). **Hoang Dang Khoa Do:** conceptualization (equal), methodology (equal), supervision (equal), writing – review and editing (equal).

## Funding

This work was supported by Tra Vinh University (TVU) under grant contract number 60/2025/HĐ.HĐKH&ĐT—ĐHTV.

## Conflicts of Interest

The authors declare no conflicts of interest.

## Data Availability

The complete chloroplast genomes of *Homalomena perplexa* were submitted to GenBank under accession numbers PX569150 (https://www.ncbi.nlm.nih.gov/nuccore/PX569150). The raw data of *Homalomena perplexa* were submitted to the Sequence Read Archive of NCBI with a BioProject accession of PRJNA1377832, BioSample accession of SAMN53801875, and SRA accession of SRR36371448.
